# Developmental Dysplasia of the Hip (DDH): Etiology, Diagnosis, and Management

**DOI:** 10.7759/cureus.43207

**Published:** 2023-08-09

**Authors:** Khaled Bakarman, Abdulmonem M Alsiddiky, Mohamed Zamzam, Kholoud O Alzain, Fahad S Alhuzaimi, Zulqurnain Rafiq

**Affiliations:** 1 Pediatric Orthopaedics, King Saud University, Riyadh, SAU; 2 Pediatric Orthopedics & Spinal Deformities, Research Chair of Spinal Deformities, King Saud University, Riyadh, SAU; 3 Pediatric Orthopedics, King Saud University, Riyadh, SAU; 4 Orthopedic Surgery, King Saud University, Riyadh, SAU; 5 Orthopedics, King Saud University, Riyadh, SAU; 6 Orthopaedics, King Saud University, Riyadh, SAU

**Keywords:** hip dysplasia review, developmental dysplasia of the hip, hip subluxation, osteotomy, congenital dislocation of the hip

## Abstract

Developmental dysplasia of the hip (DDH) is a complex disorder that refers to different hip problems, ranging from neonatal instability to acetabular or femoral dysplasia, hip subluxation, and hip dislocation. It may result in structural modifications, which may lead to early coxarthrosis. Despite identifying the risk factors, the exact aetiology and pathophysiology are still unclear. Neonatal screening, along with physical examination and ultrasound, is critical for the early diagnosis of DDH to prevent the occurrence of early coxarthrosis. This review summarizes the currently practised strategies for the detection and treatment of DDH, focusing particularly on current practices for managing residual acetabular dysplasia (AD). AD may persist even after a successful hip reduction. Pelvic osteotomy is required in cases of persistent AD. It could also be undertaken simultaneously with an open hip reduction. Evaluation of the residual dysplasia (RD) of the hip and its management is still a highly active area of discussion. Recent research has opened the door to discussion on this issue and suggested treatment options for AD. But there is still room for more research to assist in managing AD.

## Introduction and background

Developmental dysplasia of the hip (DDH) is a complex disorder that refers to different hip problems, including neonatal instability, acetabular, or femoral dysplasia, hip subluxation, and hip dislocation [1-3]. DDH has replaced the previous term 'congenital dislocation of the hip (CDH)' as several manifestations of DDH may not be detectable at the time of birth and may appear at a later stage [4]. Also, "congenital" has been replaced by "developmental" because the spectral range of the disease extended from acetabular dysplasia (AD) to complete dislocation [5].

Early detection and treatment are critical for improving pediatric quality of life. Delayed diagnosis and treatment at a later stage entail extensive surgery, which comes with greater difficulties and a worsened functional outcome [6]. Untreated dysplasia may lead to severe discomfort, pain, and osteoarthritis, requiring total hip arthroplasty [7]. Management is significantly influenced by the patient’s age and the severity of the dysplasia. The focus is on obtaining a concentric femoral head reduction and promoting acetabular and proximal femur development. A Pavlik harness or rigid abduction is used as the first step in early treatment for children. Patients who do not respond to brace treatment or present late require closed or open reduction (OR) and Spica casting. AD may persist even after a successful hip reduction. Pelvic osteotomy is required in cases of persistent AD. It could also be done simultaneously with an open hip reduction [2,5]. Evaluation of the residual dysplasia (RD) of the hip and its management is still a highly active area of debate. Recent research has provided insight into this issue and suggested treatment options for AD. But there is still room for further research to contribute to the better management of AD. This review describes the epidemiology, etiopathogenesis, and diagnosis of DDH and summarizes the current trends in managing recurrent AD.

## Review

Anatomy

Normal Hip

The hip joint is made up of the acetabulum and proximal femur. The joint is comprised of the capsule, teres ligament, transverse ligament, and pulvinar. The acetabulum is a hemispherical complex structure in growing children formed by the pubis, ischium, and ilium. The acetabular outer surface is formed by horseshoe-shaped articular cartilage. The cartilage of the acetabulum continues medially as the triradiate cartilage, and together they form the acetabular cartilage complex [8]. The labrum is attached to the outer edge of the acetabulum, thereby increasing the acetabular depth and helping to keep the hip stable [9]. If the head of the femur is not directly connected to the acetabulum, the latter does not develop properly, becoming flat in its shape [10]. At birth, the proximal femur is entirely made up of cartilage. The cephalic nucleus begins to ossify at the age of six months, while the ossification of the trochanteric nucleus is initiated at the age of five to six years [11].

Dysplastic Changes in Hip

The growth changes affect all structures in the acetabulum, proximal femur, and soft components of the dysplastic hip. The aberrant pressure exerted on the labrum by a dislocated or subluxated femoral head promotes fibrocartilage hypertrophy and the formation of fibrous tissue. A labral inversion may be present in dislocated hips, which makes reduction difficult. The limbus, which could be everted or inverted, is the thickened labrum. In some cases, the hyaline cartilage in the acetabulum thickens in the posterosuperior region of the articular cartilage forming a crest, termed a neolimbus [12]. The neolimbus develops due to eccentric pressure exerted by the femur head, which is divided into two cavities: the primary acetabulum on the medial aspect and the secondary acetabulum present laterally. When the hip is reduced, the neolimbus disappears [13]. Several abnormalities are seen in the proximal femur, including a shortened femoral neck and a delay in the development of secondary ossification. The valgus and anteversion of the dysplastic femur are exaggerated. However, there is disagreement regarding femoral anteversion between the affected and unaffected sides [14].

Natural history

The term DDH may refer to one of four clinical patterns, including hip instability, AD, hip subluxation, and dislocation [3]. The Barlow and Ortolani maneuvers show that hip dysplasia produces instability in the first few months after birth. Hip instability is common in infants, with a prevalence of 1% to 1.5% and an incidence rate of 5 per 1,000 in boys and 13 per 1,000 among girls. A spontaneous improvement is observed in approximately 90% of children with mild instability during the first two months of life [15]. This spontaneous resolution is caused by a reduction in relaxin levels and an increase in muscle tone. Only 1.2% of neonatal hip instability occurrences necessitate surgical intervention [16]. Persistent DDH left untreated results in a sequence of anatomical alterations that alter the joint biomechanics by raising tension on a reduced-contact articular surface. The maintenance of increased articular pressures for lengthy periods promotes articular cartilage degradation and early coxarthrosis. However, there is a well-established link between AD and coxarthrosis [17]. On the other hand, in the case of subluxation coxarthrosis nearly always develops in the 30s and 40s for such patients [18]. In true dislocation, whether unilateral or bilateral, it depends on whether the femoral head articulates with the ilium or not. In the bilateral case, where the femur head has not articulated with the ilium, the individuals have pain-free, excellent range-of-motion, but they have a waddling gait, hyperlordosis, and back pain. If the femoral head articulates with the ilium at any point, these patients develop disabling degenerative joint disease and require arthroplasty very early in life. Patients with unilateral dislocation develop leg length discrepancy, an unsteady gait, valgus deformities of the knee, lateral compartment degenerative joint disease, and possibly secondary scoliosis.

Etiology and pathogenesis

The optimal growth of the hip joint depends upon two main factors: first, the concentric reduction of femoral head, and second, adequate balance of growth between acetabular and triradiate cartilages. Any imbalance in these, whether during fetal development or postnatal growth, will result in abnormal hip development. The dynamic femoral and acetabular interactions are crucial in the development of hip joint. The complex nature of this condition is due to a mix of genetic, environmental, and mechanical factors. Various etiological theories of DDH have been proposed in the literature, highlighting hormonal, mechanical, and genetic factors.

Risk Factors

The hormonal theory: The hormonal theory has a significant role in hip dysplasia development. It is based on an imbalanced ratio of estrogen to progesterone. A progesterone-rich environment can promote dislocation, whereas an estrogen-rich environment can inhibit it [15].

Fetal packaging deformity: The mechanical factors are usually related to restricted space in utero resulting in fetal packaging deformities. This could be seen in the first baby. The baby may be growing inside a horn of a bicornuate uterus where there is limited space. If the baby is relatively large, there may be a subsequent packaging deformity [12].

 Breech delivery: One of the most important mechanical factors that may be a risk for DDH is breech presentation at birth. A 25% risk of DDH exists for neonates born after being in the breech position. About 30 to 50% of patients with DDH have a history of breech delivery. During breech delivery, the hips and the knees are quite extended, and the subsequently increased flexion results in the contraction of the iliopsoas muscle, thereby further dislocating the joint [19].

Swaddling: In a newborn infant, the normal hip posture is flexion and abduction. The maintenance of acetabulo-femoral contact promotes hip growth. Although the majority of AD identified in neonatal hip ultrasound recovers spontaneously, swaddling may promote deformity in infants. DDH is more likely in situations where swaddling is a common practice [20]. Swaddling has gained popularity in several developed countries in recent years due to its benefits for improving newborn sleep. The traditional infant wrapping with the lower limbs extended and adducted among Saudi population has been proposed as a predisposition to hip dislocation and future progression to an unstable hip joint [21].

Familial predisposition: An inherited predisposition has been well-established in the literature. First-degree relatives have a 12 times greater risk of acquiring a DDH, but second-degree relationships have a relative risk of only 1.7 times. In cases of DDH familial aggregation, changes in genes such as CX3CR1 have been detected [22].

Hundt et al., in a meta-analysis, emphasized that only breech presentation, females, clicking hips during the examination, and being in a familial aggregation were found to increase the chance of developing DDH [23]. However, the majority of DDH patients and those who require treatment often do not exhibit any risk factors other than being female [24].

Diagnosis

Clinical Examination

**Figure 1:**
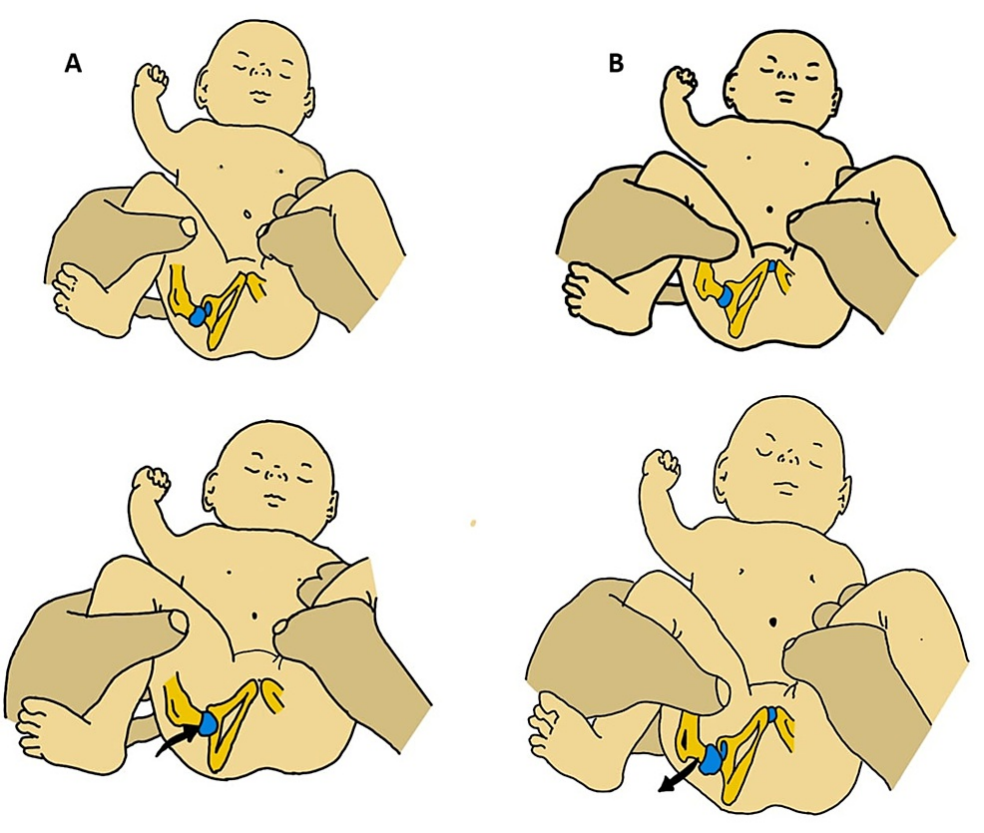
Tests for hip instability or dislocation in the newborn infant: (A) Ortolani's test; (B) Barlow's provocative test

In newborns: All neonates, in particular those displaying the risk factors for DDH, should go through a thorough clinical assessment. The Ortolani test and the Barlow maneuver should both be included in routine screening, and each hip should be checked separately for instability [25,26]. For the physical examination, the infant should be laid down on a flat, warm surface in a quiet environment. In the Ortolani reduction test, the newborn should be placed in the supine position with hip flexion kept at 90 degrees (Figure 1A). The examiner should then place his index and middle fingers on the lateral aspect of the baby's greater trochanter, while keeping his thumb medially at the groin crease. Thereafter, the stabilization of the pelvis is maintained by keeping the contralateral hip steady while the other hip is being evaluated. At the same time, an upward push is exerted through the greater trochanter laterally. Sensing a clunk is considered to be a positive result for the Ortolani test, indicating a dislocated and reducible hip. In the Barlow dislocation test, the first step is stabilizing the pelvis. The patient's position is maintained similarly to that for the Ortolani test, with the knee adducted. Then, a gentle downward force is exerted longitudinally along the femoral axis, identifying any possible posterior subluxation or dislocation by producing a palpable sensation (Figure 1B).

In older children: Examination of the extremities of infants and toddlers involves meticulous assessment of skin folds and/or discrepancies along the length of the legs that may occur in unilateral hip dislocation cases. Asymmetrical limitation abduction may also aid in the identification of children with hip dislocation. Hip dislocation can also be detected by a positive Galeazzi sign [27]. It is performed by laying the child in a supine position with the hips and knees flexed. An unequal height of the knees indicates a positive test. In neglected cases, when children reach walking age, they limp on the affected side, resulting in a positive Trendelenberg sign and hyperlordosis.

Imaging

Ultrasonography: Because the head of the femur and acetabulum are predominantly composed of cartilage, standard radiographs have poor diagnostic value in neonates [28]. Ultrasonography is the investigation of choice for DDH in the first six months of life. It is more beneficial to evaluate subtle sub-types of the disorder when the clinical examination is inconclusive. Moreover, this is the only imaging mode that provides real-time 3D images of the hip joints of newborns. Other benefits include the avoidance of radiation, hip joint puncture, medium contrast, and sedation. It provides a detailed evaluation of the cartilaginous femoral head and demonstrates the relationship of the head to the bone as well as the cartilaginous acetabulum [28]. Graf et al. developed a strategy based on the morphological features of the hip, requiring the calculation of two angles: the alpha angle, between the ilium and the osseous acetabular wall, and the beta angle, between the ilium and the labral cartilage [29].

**Figure 2:**
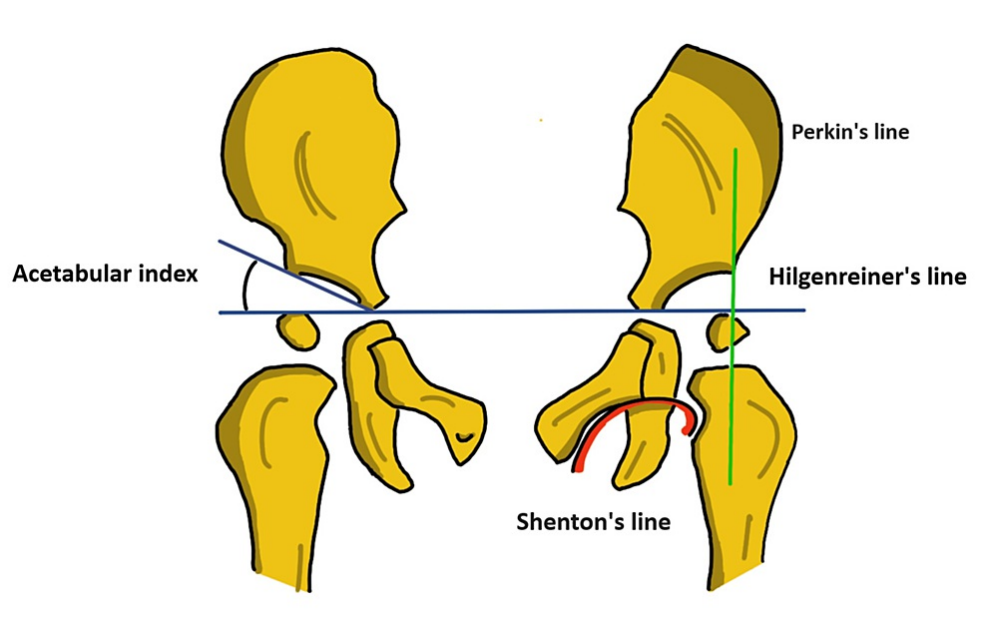
Reference lines and angles used to evaluate in DDH

Radiography: The radiographic examination is a more useful method of evaluating hip development. Several classic lines on the X-ray of the immature pelvis guide the process of assessing DDH (Figure 2) [30]. Hilgenreiner's line is a line joining both of the triradiate cartilages. The Perkins line extends along the lateral border of the acetabulum and is at right angles to the Hilgenreiner's line in a normal hip. The Shenton’s line contains a curvature that starts at the lesser trochanter, extends upwards towards the neck of the femur, and connects to a line along the inner margin of the pubis. In a normal hip, Shenton's line is smooth. This line is non-continuous when the affected hip is subluxated or dislocated. The angle formed at the intersection of Hilgenreiner’s line and the line drawn along the surface of the acetabulum is called the acetabular index. As the baby grows, this angle changes as well. It measures how much the roof of the acetabulum is inclined. This is the most frequently employed parameter in assessing the morphological features of the acetabulum. In normal newborns, this angle is 27.50 degrees, 23.50 degrees at six months, and progresses to 20 degrees at second birthday. Generally, 30 degrees is considered as normal upper limit and a notable increase in this value is considered a sign of AD [31].

**Figure 3:**
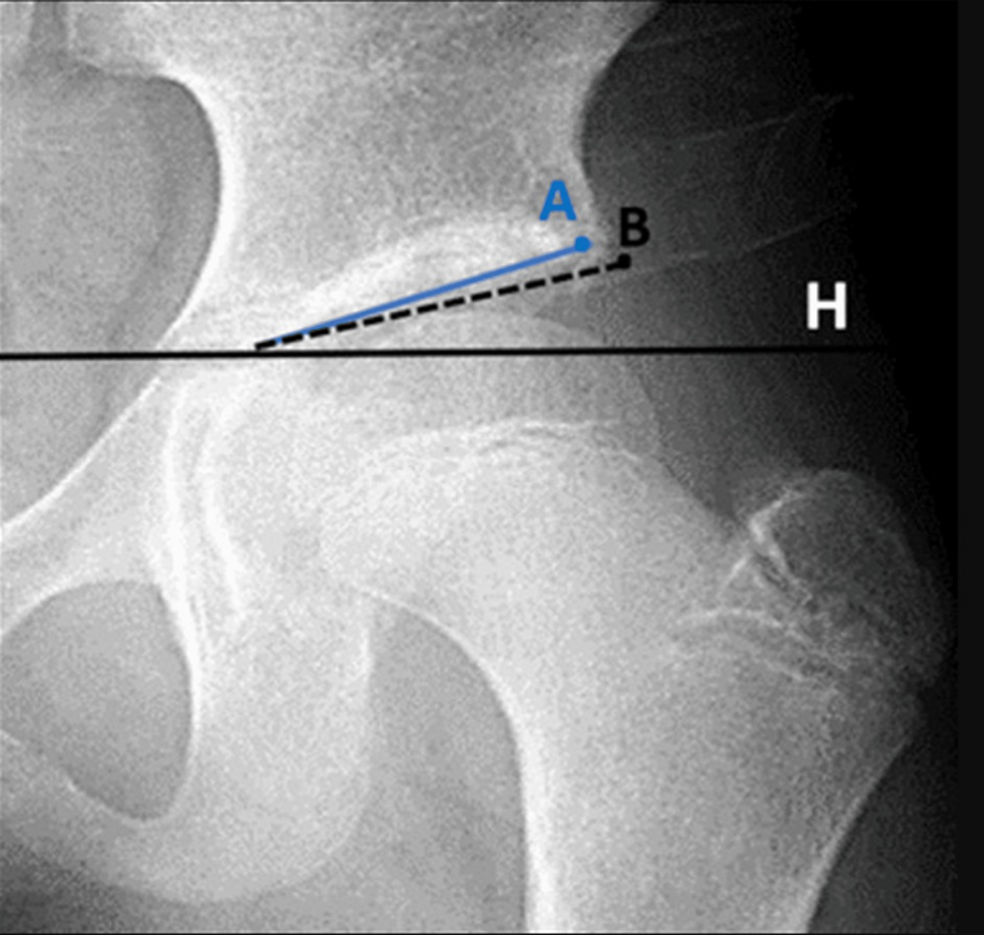
Two different measuring reference points. Novais et al. used the lateral edge of the weight-bearing sourcil (point A), while Tonnis used the lateral bony edge (point B). H indicates Hilgenreiner's line.

However, it is important to consider the variations in normal indices among the various research papers. Moreover, the method used for the measurement of the acetabular index should be noted. Novais et al. and Tonnis both positioned the horizontal "Hilgrenreiner line" at the lower lateral iliac edge on the triradiate cartilage [28,32]. Novais et al. selected the lateral margin of the weight-bearing sourcil, whereas Tonnis used the lateral bony margin as shown in Figure 3. However, disagreements on the landmark for the lateral margin persist.

The Wiberg centre-edge angle (CEA) is formed by the Perkin's line and the line from the centre of the femoral head to the lateral acetabulum. Due to the difficulty in locating the centre of the head of the femur, it exhibits significant variability in the initial three years of life. Among older children, it measures how much of the acetabulum covers the head of the femur. In children aged 3 to 17 years, an abnormal angle is one less than 15 degrees. On the other hand, angles greater than 25 degrees are categorized as normal among adults, while values under 20 degrees are regarded as abnormal [32]. Severin's classification evaluates the hip at maturity with a good correlation regarding long-term radiological, clinical, and functional hip results [33].

CT and MRI: CT is also among the imaging modalities used to assess reduction quality after closed or OR in a Spica cast [34]. CT contributes towards evaluating dysplasia in adolescents and young adults and allowing for better selection of the type of surgery required, such as pelvic or femoral osteotomies. A limited CT emits low ionizing radiation, although MRI is now successfully employed to eliminate radiation exposure [35]. MRI is considered as a predictor of AVN after closed reduction in DDH. In addition, MRI is also a useful tool in detection and assessment of labral abnormalities [35].

Arthrography: Arthrography is beneficial in the non-ossified skeleton because it facilitates the assessment of soft tissues and cartilages of the femoral head and acetabulum. As a result, it is frequently utilized as an intraoperative dynamic test to determine the quality of reduction and hip joint stability. It is critical in determining whether to use closed or OR [36].

Treatment of DDH

**Figure 4:**
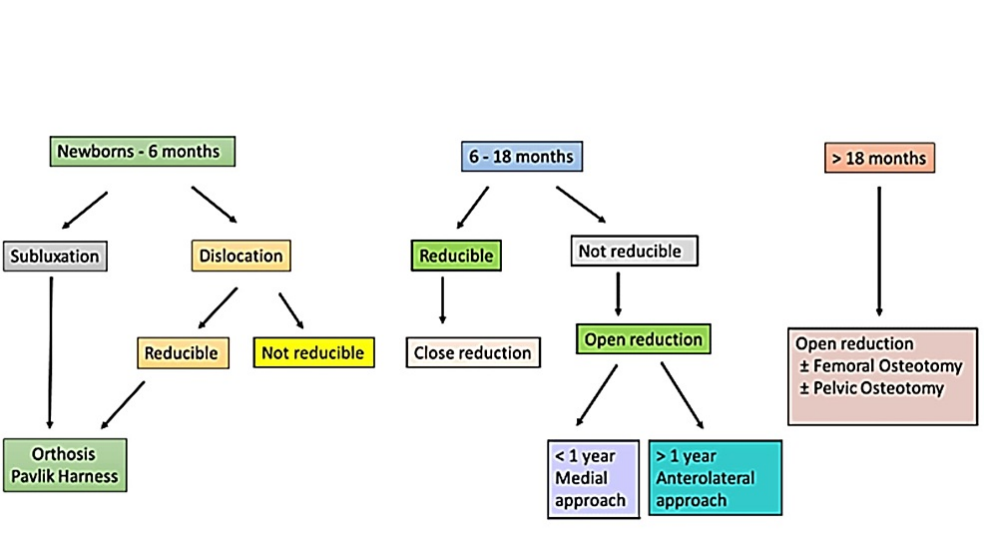
Treatment algorithm for DDH according to age DDH: Developmental dysplasia of the hip

The aim of DDH treatment depends on the patient's age at the time of diagnosis and requires concentric reduction of the femoral head into the acetabulum (Figure 4) [37,38].

Newborn to six months of age

Patients should ideally be diagnosed and managed during infancy. Hip subluxation, which is usually resolved spontaneously, can be observed for three weeks without any treatment. The commencement of treatment is recommended after three weeks if the evidence of subluxation on physical and ultrasonographic assessment is present [2]. When the hip joint is fully dislocated at the neonatal stage, it is advised to start treatment immediately. Hip reduction is easier, and the Pavlik harness is the most often used orthosis during this period. In most cases, hip reduction enables the acetabulum to normalize itself during this age. In the 1940s, Arnold Pavlik invented the "harness with stirrups" [38,39]. When the hip and knee are flexed with the hip in an abducted position while dynamic hip movements are enabled, the hip adduction contraction will relax and subsequently reduce spontaneously during abduction motions [2,38]. It is recommended to wear the Pavlik harness with the hips abducted between 30° and 60°. The major objective is to achieve spontaneous and painless realignment and to centralize the femoral head in children from neonatal age until the age of six to 10 months to achieve optimal structural and functional outcomes [2].

The Pavlik harness had a 95% success rate in cases of AD or hip subluxation and an 80% success rate in cases of hip dislocation [2]. The Pavlik harness is the most widely used approach in managing pediatric DDH from birth to six months, according to various publications, and it remains the standard treatment [27,36,38]. It is safe and extremely effective. Residual AD still poses a substantial challenge after the orthopaedic intervention. Another study reported that after successfully using closed reduction with the Pavlik harness, about 30% of patients had AD [38,40]. The harness is associated with a few complications that occur rarely if it is used appropriately. Avascular necrosis (AVN) of the femoral head is reported as the most serious complication and is associated with excessive abduction of the hip. Placing the harness such that the hips are flexed excessively may dislocate the joint in a downward direction or even result in femoral nerve palsy [2].

Persistent dysplasia or instability between six and 18 months

With increasing age, the hip reduction becomes more challenging and decreases the effectiveness of the Pavlik harness. If the hip reduction fails or the child is older than six months, then this is an indication for a closed or OR and Spica cast immobilization. Dynamic arthrography using fluoroscopy is recommended for evaluating the reduction quality to determine whether the reduction should be closed or open [2,5,9].

Closed Reduction

For children older than six months, a closed reduction and spica cast immobilization is indicated under general anaesthesia with the hips flexed at 90 to 100 degrees with well-controlled abduction. Immobilization should not be performed in an excessive hip abduction position. Serial radiographs are used to monitor hip development. It has been reported that the majority of patients who achieved successful closed reduction may require additional treatment after 18 months, as a sizable number of individuals had persistent AD, necessitating future acetabular osteotomies [41]. Forced close reduction in the presence of interposed structures leads to poor outcomes and an elevated risk for AVN [42].

Open Reduction

With age, the risk of OR increases. OR is recommended when closed reduction has failed to reduce the dislocated hip into a stable, concentric position. Although OR is challenging, concentric reduction promotes normalization in AD because of its growth potential [37]. Once OR is achieved, maintenance with a cast for three months facilitates hip stabilization.

Older than 18 months

When the hip dislocation is not detected early, secondary alterations take place in the soft tissues around the joint and subsequently in the proximal femur and the acetabulum. AD may still occur even if the reduction is carried out within the first few months of life. The potential of a dysplastic acetabulum to become normal diminishes with age [2,15]. Up to 19% of patients who had successful treatment with the Pavlik harness developed RD. Similar to this, persistent dysplasia may occur in 22% to 33% of patients who have had a closed or OR [43,44]. The age of the patient at the time of the surgery may have an impact on this variability [2,15]. With persistent hip dislocations, significant secondary adaptive alterations exacerbate the pathophysiology of hip dysplasia. Surgery is usually required to reconstruct the acetabulum and the femur, and the release of periarticular soft tissues is usually necessary for older children. When indicated, reconstruction measures may include a pelvic or femoral osteotomy [2,43].

Femoral osteotomies can facilitate reduction by shortening and reorienting the femoral head by derotation [45]. Osteotomy increases the varus of the hip joint to stabilize and stimulate acetabular growth and a reduction in the rate of osteonecrosis. These techniques are based on the controversial concepts of coxa valga and increased femoral anteversion. Subluxation of the hip is frequently believed to recur because of femoral anteversion, necessitating derotational osteotomy to maintain a stable hip reduction [40]. The indications for femoral derotational osteotomy are still unclear due to a lack of consensus. Although previous research suggests the common use of derotational osteotomy, studies done recently do not recommend this [46]. It has recently been recommended that, because of the inconsistency of femoral anteversion derotation osteotomy be performed on a case-by-case basis [46].

**Figure 5:**
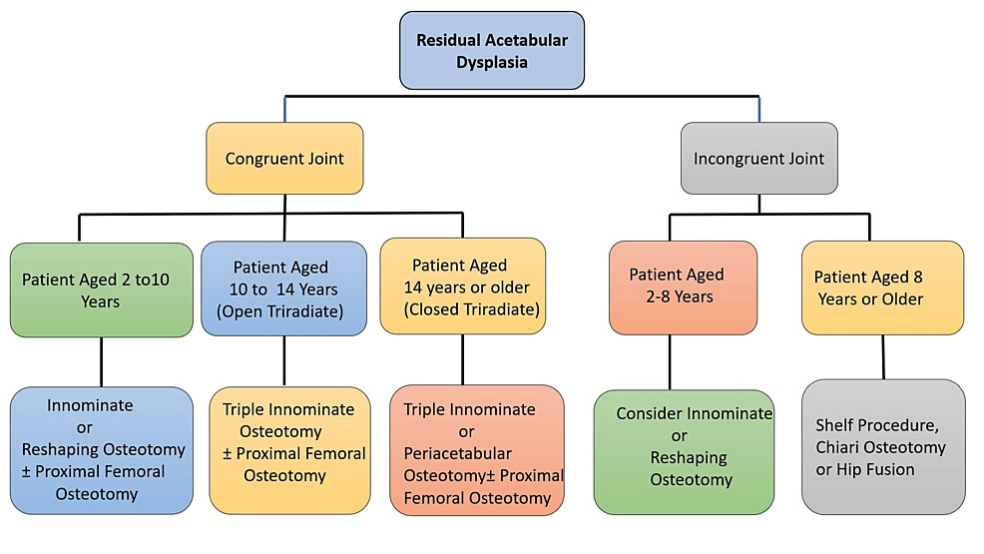
Treatment algorithm for residual AD AD: Acetabular dysplasia

Pelvic osteotomy is recommended in cases where AD persists or is detected later in its development. Pelvic osteotomy facilitates the process by increasing the cover of the femoral head on the acetabular side. In recent years, there has been a trend to perform an acetabular intervention during primary treatment to optimize the chances of normal acetabular development [2,40,43,47]. Pelvic osteotomies can be organized into three subsets based on their intended effect on the acetabular morphology (Figure 5).

Redirectional Osteotomies 

**Figure 6:**
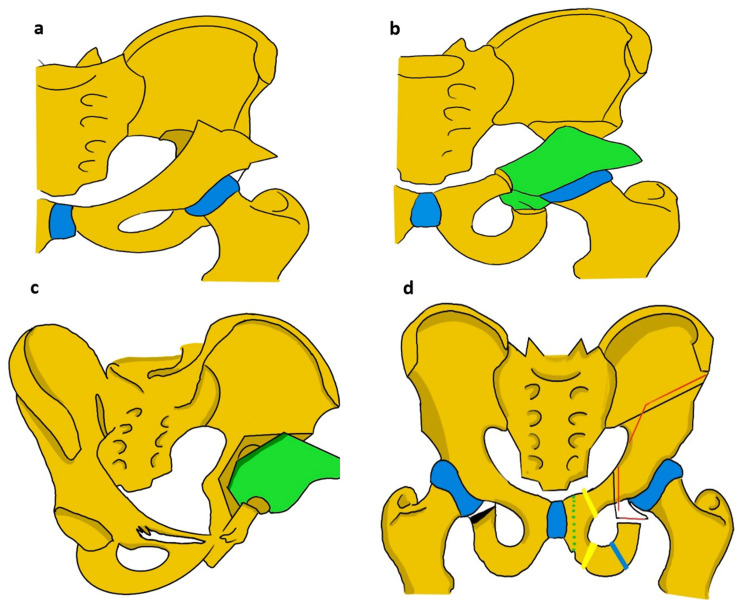
Redirection osteotomies (a) Salter’s osteotomy; (b) TIO; (c) periacetabular osteotomy; (d) evolution of the TIO, modification of ischial cuts, southerland (green dotted line); Carlioz (orange lines in pubic and ischial bones ); Steel’s (blue line); and Tonnis TIO (black lines) Bernes TIO (red line) TIO: Triple innominate osteotomy

Re-directional pelvic osteotomy shifts the position of the acetabulum while leaving its shape and volume unchanged [2,36,43,38]. Because these osteotomies are performed via complete cuts of the innominate bone, they are unstable and require stabilization with internal fixation. The three most commonly performed redirection osteotomies are the Salter, triple, and periacetabular osteotomies, commonly referred to as periacetabular osteotomy (PAO) (Figure 6).

Reshaping Osteotomies

**Figure 7:**
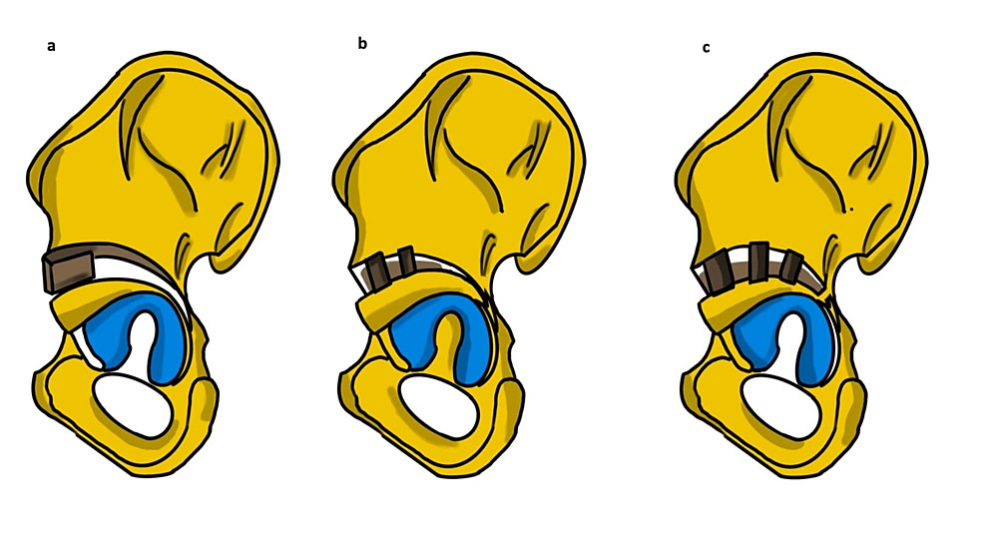
Reshaping osteotomies (a) Pemberton; (b) Dega; (c) San Diego osteotomies as viewed from the outer surfaces of the ilium

Reshaping osteotomies are ultimately aimed at achieving a congruently reduced femoral head and acetabulum [47]. These osteotomies are incomplete innominate osteotomies and are associated with high correction rates of AD as shown in Figure 7. The objective of these osteotomies is to restore the acetabular morphology by changing the shape of a capacious and wandering acetabulum. The osteotomies consist of an incomplete opening wedge osteotomy in the peri-acetabular area held open with a bone graft that results in a change in the acetabular slope, shape, and volume. These are appropriately referred to as "acetabuloplasties" and are inherently stable; therefore, fixation is not necessary. The size, direction, and location of the opening wedge dictate the resulting change and acetabular coverage. These osteotomies rely on hinges through the triradiate cartilage and are therefore indicated only in skeletally immature patients. Three of the most commonly performed reshaping osteotomies are the Dega, San Diego, and Pemberton osteotomies. These osteotomies are quite similar in their approach and vary slightly concerning the extent to which the inner table is cut and how close the osteotomy is to the joint [43,48].

Salvage/Augmentation Procedures

Salvage osteotomies are utilized when concentric reduction may not be possible. The goal is to merely increase the weight-bearing surface of the hip. Numerous factors influence the choice of pelvic osteotomy in cases of DDH, including the surgeon's preference, the patient's age, and skeletal maturity, as well as the congruity, morphological features, and volume of the hip joint itself [2,47].

**Figure 8:**
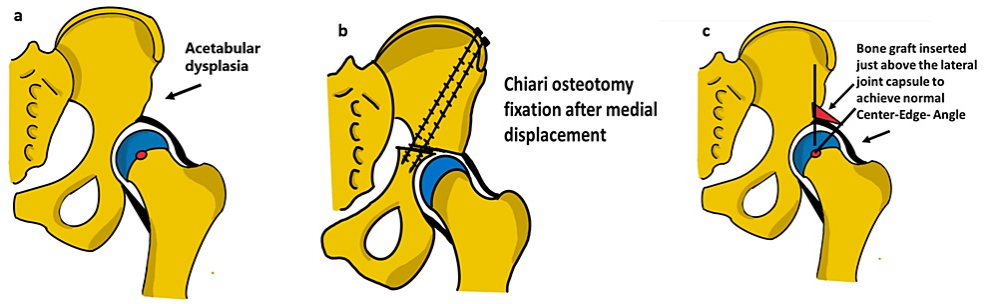
Schematic illustration (a) a dysplastic acetabulum with deficient superior coverage; (b) the Chiari osteotomy; (c) shelf acetabuloplasty both improves superior coverage

Salvage osteotomies are recommended in cases where the femoral head and the acetabulum may not be congruently reduced or where hyaline cartilage is insufficient for femoral head coverage. Such an osteotomy may also be appropriate in cases with a painful subluxated hip or previous failed surgical interventions [2, 36, 48]. These procedures aim to increase the weight-bearing surface area of the hip by causing metaplasia of hip capsular tissue into fibrocartilage. Two commonly utilized salvage procedures for the hip are the Chairi and Shelf osteotomies as depicted in Figure 8.

## Conclusions

Early diagnosis of DDH is critical for a favorable outcome. Newborns with DDH should be managed with a Pavlik harness until the age of six months. For children who have failed Pavlik harness treatment, a closed or OR is recommended. 18-month-old children should be treated by OR along with femoral or pelvic osteotomies where indicated. Post-surgical radiological follow-up is mandatory for residual AD, which is a frequently encountered complication of DDH treatment. Surgeons should highlight the importance of counselling parents about the possibility of RD and the requirements of additional surgeries following closed or OR. The decision to perform additional procedures depends on the patient's age, level of dysplasia, skeletal maturity, and acetabular deficiency.
